# Effects of 28 days of resistance exercise while consuming commercially available pre- and post-workout supplements, NO-Shotgun^® ^and NO-Synthesize^® ^on body composition, muscle strength and mass, markers of protein synthesis, and clinical safety markers in males

**DOI:** 10.1186/1743-7075-8-78

**Published:** 2011-11-03

**Authors:** Mike Spillane, Neil Schwarz, Sarah Leddy, Tracie Correa, Melodie Minter, Victoria Longoria, Darryn S Willoughby

**Affiliations:** 1 Department of Health, Human Performance, and Recreation, Baylor University, Box 97313, Waco, TX76798, USA; 2 Institute for Biomedical Studies, Baylor University, Waco, TX87898, USA

## Abstract

**Purpose:**

The effects of 28 days of heavy resistance training while ingesting the pre- and post-workout supplements, NO-Shotgun^® ^and NO-Synthesize^® ^were determined on body composition, muscle strength and mass, markers of protein synthesis, and clinical safety markers.

**Methods:**

Nineteen non-resistance-trained males participated in a resistance training program 4 times/week for 28 days while either ingesting 27 g/day of carbohydrate (CARB) or NO-Shotgun^® ^30 min pre-exercise and 27 g/day of carbohydrate or NO- Synthesize^® ^30 min post-exercise (NOSS). Data were analyzed with separate 2 × 2 ANOVA (p < 0.05).

**Results:**

Total body mass was increased in both groups (p = 0.001), but not different between groups. Fat mass was unchanged with CARB, but NOSS decreased fat mass (p = 0.026). Both groups increased fat-free mass (p = 0.001); however, the increases were greater with NOSS (p = 0.023). NOSS underwent greater increases in upper-body (p = 0.023) and lower-body (p = 0.035) strength than CARB. Myofibrillar protein significantly increased in both groups (p = 0.041), with NOSS being greater than CARB (p = 0.049). All of the MHC isoforms were significantly increased in both groups; however, NOSS was greater than CARB for MHC 1 (p = 0.013) and MHC 2A (p = 0.046). All of the myogenic regulatory factors were significantly increased in both groups; however, NOSS was greater than CARB for Myo-D (p = 0.038) and MRF-4 (p = 0.001). For the whole blood and serum clinical chemistry markers, all variables remained within normal clinical ranges.

**Conclusions:**

Heavy resistance training for 28 days, with NO-Shotgun^® ^and NO-Synthesize^® ^ingested before and after exercise, respectively, significantly improved body composition and increased muscle mass and performance without abnormally impacting any of the clinical chemistry markers.

## Introduction

Heavy resistance training augments muscle protein synthesis [1-3], thereby resulting in increases in muscle strength and hypertrophy [4-6]. It has been suggested that the ingestion of specific nutrients (e.g., protein, amino acids, carbohydrate, creatine, etc.) [7-10], or a combination of nutrients (i.e., protein+carbohydrate, protein+carbohydrate+creatine, protein+amino acids, etc.) [11-13] within approximately one hour before and/or after resistance exercise will augment substrate availability that is necessary during exercise and several hours into the recovery period. The ingestion of either protein or creatine before and after resistance exercise for 16 weeks was shown to be more effective in increasing muscle strength and satellite cell activation than resistance training without nutrient provision [9]. We have shown that ingesting protein (whey and casein) and amino acids before and after resistance exercise for 10 weeks resulted in significantly greater increases in muscle strength and mass compared to iso-caloric carbohydrate [13].

As a result, many recent studies have chosen to provide nutrients in close proximity (either before and/or after) to resistance exercise [11-14]. This concept of nutrient timing has been demonstrated in a 10 week-study in which a supplement comprised of protein, creatine, and glucose was given immediately before and after each resistance exercise session or in the morning and evening. Providing the supplement before and after exercise resulted in a greater improvement in muscle strength and mass, Type II muscle fiber cross-sectional area, and contractile protein content [14]. However, more recently it was shown that a protein supplement provided before and after resistance exercise for 10 weeks was no more effective at increasing muscle strength and mass compared to when the protein supplement was provided in the morning and evening [7]. As such, there appears to be disagreement in the literature regarding this nutritional timing strategy during resistance training, yet it continues to be considered to be a more effective method of bolstering increases in muscle mass and strength compared to resistance training without pre- and/or post-exercise nutrient provision.

Recently we conducted a study to determine the effects of an alleged pre-workout supplement and demonstrated that four weeks of heavy resistance training in conjunction with the provision of the nutritional supplement, NO-Shotgun^®^, 30 min prior to each exercise session was more effective at increasing muscle strength and mass and markers indicative of muscle protein synthesis and satellite cell activation when compared to carbohydrate [15]. Based on our previous study [15], and using the same experimental design in the present study, we wanted to also provide a nutritional supplement post-exercise to compare the effects compared to carbohydrate.

Therefore, the purpose of this study was to compare the effects of four weeks of heavy resistance training performed in conjunction with either carbohydrate or NO-Shotgun^® ^before and NO-Synthesize^® ^after each exercise session on muscle strength, body composition, markers of protein synthesis, and clinical safety markers in men.

## Methods

### Participants

Nineteen apparently healthy, recreationally active, non-resistance trained [no consistent (at least thrice weekly) resistance training for one year prior to the study] males with an average age of 22.8 ± 4.67 yr, height of 179.5 ± 6.38 cm, and total body mass of 79.1 ± 16.13 kg completed the study. Enrollment was open to men of all ethnicities. All participants passed a mandatory medical screening. Participants with contraindications to exercise as outlined by the American College of Sports Medicine and/or who had consumed any nutritional supplements (excluding multi-vitamins) such creatine monohydrate, nitric oxide-stimulating, hydroxy-beta-methylbutyrate (HMB), various androstenedione derivatives, or pharmacologic agents such as anabolic steroids three months prior to the study were not allowed to participate. All eligible participants signed a university-approved informed consent document based on the guidelines set forth by the Institutional Review Board for the Protection of Human Subjects of Baylor University. Additionally, all experimental procedures involved in this study conformed to the ethical considerations of the Helsinki Code.

### Testing sessions

The study included baseline testing at day 0 and a follow-up testing session at day 29 in which blood and muscle samples were obtained and where body composition and muscle performance tests were performed.

### Strength assessment

Upper- and lower-body one repetition maximum (1-RM) strength tests were performed using the free weight bench press and angled leg press exercises (Nebula, Versailles, OH), respectively. Initially, an estimated 50% 1-RM was utilized to complete 5 to 10 repetitions. After a two min rest period, a load of 70% of estimated 1-RM was utilized to perform 3 to 5 repetitions. Weight was gradually increased until a 1-RM was reached with each following lift, with a two min rest period in between each successful lift. Test-retest reliability of performing these strength assessments on subjects within our laboratory during the previous year has demonstrated low mean coefficients of variation and high reliability for the bench press (1.7%, intra-class *r *= 0.92) and leg press (0.72%, intra-class *r *= 0.93), respectively.

### Body composition assessment

Total body mass (kg) was determined on a standard dual beam balance scale (Detecto Bridgeview, IL). Percent body fat, fat mass, and fat-free mass were determined using DEXA (Hologic Discovery Series W, Waltham, MA). Quality control calibration procedures were performed on a spine phantom (Hologic X-CALIBER Model DPA/QDR-1 anthropometric spine phantom) and a density step calibration phantom prior to each testing session. The DEXA scans were segmented into regions (right & left arm, right & left leg, and trunk). Each of these segments was analyzed for fat mass, lean mass, and bone mass. Based on previous quality control testing in our laboratory from the previous year, the accuracy of the DEXA for body composition assessment is ± 2.3% as assessed by direct comparison with hydrodensitometry and scale weight. Total body water volume was determined by bioelectric impedance analysis (Xitron Technologies Inc., San Diego, CA) using a low energy, high frequency current (500 micro-amps at a frequency of 50 kHz).

### Venous blood sampling and percutaneous muscle biopsies

Venous blood samples were obtained from the antecubital vein into a 10 ml collection tube using a standard vacutainer apparatus. Blood samples were allowed to stand at room temperature for 10 min and then centrifuged. The serum was removed and frozen at -80°C for later analysis. Percutaneous muscle biopsies (50-70 mg) were obtained from the middle portion of the vastus lateralis muscle of the dominant leg at the midpoint between the patella and the greater trochanter of the femur at a depth between 1 and 2 cm. After sample removal, adipose tissue was trimmed from the muscle specimens, immediately frozen in liquid nitrogen, and stored at -80°C for later analysis.

### Supplementation protocol

Participants were assigned to a 28-day supplementation protocol, in a double-blind, placebo-controlled manner. Participants ingested either 54 g/day of maltodextrose (CARB) or 27 g/day of NO-Shotgun^® ^and 27 g/day of NO-Synthesize^® ^(NOSS). For CARB, 27 g were ingested 30 min prior to exercise and 27 g within 30 min following exercise. NOSS ingested 27 g of NO-Shotgun^® ^30 min prior to exercise and 27 g/day NO-Synthesize^® ^within 30 min following exercise. Immediately upon waking on non-training days, CARB ingested 27 g of the supplement, whereas NOSS ingested 27 g of NO-Synthesize^®^. For supplementation compliance, participants completed questionnaires and returned empty containers during the post-study testing session on day 29.

### Dietary monitoring

In order to monitor dietary intake, participants were required to record their food and drink intake for four days prior to each of the two testing sessions at day 0 and day 29. For standardization purposes, participants' diets were not standardized and subjects were asked not to change their dietary habits during the course of the study. The four-day dietary recalls were evaluated with the Food Processor IV Nutrition Software (ESHA, Salem OR) to determine the average daily macronutrient intake of fat, carbohydrate, and protein for the duration of the study.

### Resistance-training protocol

Based on our previous study [15], participants completed a periodized 28-day resistance-training program split into two upper-extremity and two lower-extremity exercise sessions each week. This constituted a total of 16 exercise sessions, with eight upper-body and eight lower-body exercise sessions. Prior to each exercise session, participants performed a standardized series of stretching exercises. The participants then performed an upper-extremity resistance-training program consisting of nine exercises (bench press, lat pull, shoulder press, seated rows, shoulder shrugs, chest flies, biceps curl, triceps press down, and abdominal curls) twice per week and a program consisting of seven lower-extremity exercises (leg press or squat, back extension, step ups, leg curls, leg extension, heel raises, and abdominal crunches). Participants performed three sets of 10 repetitions at 70 - 80% 1-RM. Rest periods were two min between exercises and sets. The resistance exercise sessions were not supervised; however, it was required that each participant completed detailed daily resistance-training logs.

### Whole blood and serum clinical chemistry analyses

Whole blood was collected and immediately analyzed for standard cell blood counts with percentage differentials (hemoglobin, hematocrit, RBC, MCV, MCH, MCHC, RDW, WBC counts, neutrophils, lymphocytes, monocytes, eosinophils, basophils and leukocyte differentials) using an automated hematology analyzer (Sysmex XS-1000i, Mundelein, IL). The instrument's flow system was primed and the background counts checked daily to ensure appropriate RBC and WBC linearity. Based on the quality control standards from the manufacturer, the coefficients of variation for the Sysmex XS-1000i were 0.82%, 0.84%, 0.026%, 0.75%, and 0.82% for neutrophils, lymphocytes, monocytes, eosinophils, and basophils, respectively, and fell within the recommended ranges.

Serum samples were out-sourced (Quest Diagnostics, Dallas, TX) and assayed for general clinical chemistry markers (total cholesterol, high-density lipoproteins, low-density lipoproteins, triglycerides, albumin, glucose, GGT, LDH, uric acid, BUN, creatinine, BUN/creatinine ratio, calcium, creatine kinase, total protein, total bilirubin, ALP, ALT, and AST). Based on the methodology employed for analysis, the coefficients of variation for all analyses reported by Quest Diagnostics (Dallas, TX) were no greater than 6%.

### Serum IGF-1 analysis

Serum samples were analyzed in duplicate for IGF-1 (Enzo Life Sciences, Plymouth Meeting, PA) and HGF (R&D Systems, Minneapolis, MN) using an ELISA. For IGF-1, this assay has a sensitivity of 34.20 pg/ml and does not cross-react with IGFBPs 2, 3, and 4, HGF, or insulin. For IGF-1, the subsequent absorbances, which were directly proportional to the concentration of analyte in the sample, were measured at a wavelength of 450 nm using a microplate reader (iMark, Bio-Rad, Hercules, CA). A set of standards of known concentrations for IGF-1 was utilized to construct a standard curve by plotting the net absorbance values of the standards against the respective peptide concentrations. By applying a four-part parameter curve using data reduction software (Microplate Manager, Bio-Rad, Hercules, CA), the serum IGF-1 concentrations were calculated. The overall intra-assay percent coefficient of variation was 5.3% for IGF-1.

### Skeletal muscle cellular extraction

Each muscle sample was weighed and approximately 20 mg were homogenized using a commercial cell extraction buffer (Biosource, Camarillo, CA) and a tissue homogenizer. The cell extraction buffer was supplemented with 1 mM phenylmethanesulphonylfluoride (PMSF) and a protease inhibitor cocktail (Sigma Chemical Company, St. Louis, MO) with broad specificity for the inhibition of serine, cysteine, and metallo-proteases.

### Myogenic regulatory factor quantitation

The muscle protein expression of the MRFs was assessed through the use of ELISA [15]. Polyclonal antibodies specific for Myo-D, myogenin, and MRF-4 were purchased from Santa Cruz Biotech (Santa Cruz, CA). Initially, the antibodies were diluted to 1 μg/ml in coating buffer (Na2CO3, NaHCO3, and ddH2O, pH 9.6) and allowed to incubate at room temperature overnight. Following incubation, the plates were washed (1X phosphate buffered saline, Tween-20), blocked (10X phosphate buffered saline, bovine serum albumin, ddH2O), washed, and then incubated with a secondary antibody (IgG conjugated to HRP) diluted to 1 μg/ml in dilution buffer (10X phosphate buffered saline, Tween-20, bovine serum albumin, ddH2O). After washing, a stabilized TMB chromogen was added and the plates were covered and placed in the dark for the last 30-min prior to being stopped with 0.2 M sulphuric acid. The subsequent absorbances, which were directly proportional to the concentration of the MRFs in the samples, were measured at a wavelength of 450 nm. There were no standards used in these ELISAs, thus no standard curve was created. Therefore, the absorbances relative to muscle weight were assessed. The overall intra-assay percent coefficients of variation were 7.12%, 6.47%, and 8.03% for Myo-D, myogenin, and MRF-4, respectively.

### Myofibrillar protein content

Myofibrillar protein was isolated from the skeletal muscle cellular extracts with repeated incubations in 0.1% SDS at 50°C and separated by centrifugation. Myofibrillar protein content was determined spectrophotometrically based on the Bradford method at a wavelength of 595 nm [13,15]. A standard curve was generated (R = 0.99, p = 0.001) using bovine serum albumin (Bio-Rad, Hercules, CA), and myofibrillar protein content was expressed relative to muscle wet-weight.

### Myosin heavy chain isoform protein quantitation

The MHC protein isoform composition within 20 μg muscle homogenates was determined under denaturing conditions using an Experion Pro260 automated electrophoresis system (Bio-Rad, Hercules, CA) using the principles of SDS-PAGE and LabChip (Caliper Life Sciences, Hopkinton, MA) technology [13]. The Experion Pro260 analysis kit has a resolution and quantitation of 10-260 kDa proteins while also separating and detecting 2.5-2,000 ng/μl protein. The Experion Pro260 system combines electrophoresis, staining, destaining, imaging, band detection, and basic data analysis into a single, automated step. Gel images were then processed and displayed on a computer monitor and MHC bands identified by migration relative to the molecular weight marker (data not shown). The density of the MHC bands was determined using Experion Imaging software (Bio-Rad, Hercules, CA), expressed in arbitrary density units.

### Reported side effects from supplements

Participants reported by questionnaire at the testing session on day 29 how well they tolerated the supplementation protocol, in addition to reporting any medical problems and/or symptoms they may have encountered during the study.

### Statistical analysis

Data were analyzed with separate 2 (group) × 2 (time) ANOVA with repeated measures on the second factor with SPSS 16.0 software (SPSS inc., Chicago, IL). Significant differences among groups were identified by a Tukey HSD post-hoc test. A probability level of < 0.05 was adopted throughout.

## Results

### Subject demographics

Twenty-two participants began the study; however, three were withdrawn due to reasons unrelated to the study. One participant contracted mononucleosis and another injured his knee and neither were able to exercise for several weeks. The third participant withdrew because he did not have an adequate amount of time in his schedule to remain compliant with the resistance training program. As a result, 19 participants completed the study. The CARB group (n = 9) had an average (±SD) age of 20.00 ± 1.41 yr, height of 179.75 ± 6.22 cm, and total body mass of 81.43 ± 16.46 kg. The NOSS group (n = 10) had an age of 21.20 ± 1.98 yr, height of 178.00 ± 4.88 cm, and total body mass of 81.41 ± 29.39 kg.

### Dietary analysis, supplement compliance, and reported side effects

The diet logs were used to analyze the average daily caloric and macronutrient consumption (Table 1). Neither group significantly increased their caloric intake during the course of the study. In addition, no significant differences existed between groups for total caloric (p = 0.129), protein (p = 0.216), carbohydrate (p = 0.106), and fat intake (p = 0.665).

**Table 1:** Dietary caloric and macronutrient intake for the CARB and NOSS groups

Variable	Group	Day 0	Day 29	Test (p < .05)	Group x Test (p < .05)
**Total Calories (kcal/kg)**				.683	.129

	**NOSS**	30.12 ± 9.94	31.61 ± 10.58		

	**CARB**	41.81 ± 18.98	35.41 ± 16.16		

**Protein (g/kg)**				.763	.216

	**NOSS**	1.17 ± 0.33	1.25 ± 0.42		

	**CARB**	1.57 ± 0.77	1.31 ± 0.43		

**Carbohydrate (g/kg)**				.932	.106

	**NOSS**	3.64 ± 1.39	4.01 ± 1.41		

	**CARB**	5.03 2.82	4.13 2.22		

**Fat (g/kg)**				.551	.665

	**NOSS**	1.17 ± 0.41	1.15 ± 0.44		

	**CARB**	1.47 ± 0.64	1.27 ± 0.77		

Data are presented as means and standard deviations.

All participants appeared to have exhibited 100% compliance with the resistance training and supplementation protocol, and were able to complete the required dosing regimen and testing procedures. Over the course of the 28 days, two participants in CARB and three in NOSS reported side effects. For CARB, both participants reported feelings of nausea, one reported a rapid heart rate, and one reported shortness of breath. For NOSS, three participants reported dizziness, two reported feelings of nausea, three reported headache, two reported a rapid heart rate, one reported shortness of breath, and one reported nervousness.

### Body composition

Total body mass was significantly increased in both groups with training (p = 0.001) with no significant changes occurring in total body water (p = 0.345). Fat mass was unchanged with CARB, but NOSS decreased fat mass (p = 0.026). Both groups increased fat-free mass with training (p = 0.001); however, the increases were greater with NOSS (p = 0.023) (Table 2).

**Table 2:** Body composition and muscle strength variables for the CARB and NOSS groups

Variable	Group	Day 0	Day 29	Test (p < .05)	Group x Test (p < .05)
**Body Mass (kg)**				.010*	.793

	**NOSS**	81.41 ± 16.46	82.64 ± 15.97		

	**CARB**	84.41 ± 29.39	85.44 ± 29.32		

**Body Water (kg)**				.345	.587

	**NOSS**	43.07 ± 10.33	43.43 ± 9.68		

	**CARB**	43.18 ± 6.50	44.12 ± 7.13		

**Body Fat (%)**				.026*	.014† NOSS < PLC

	**NOSS**	17.88 ± 7.67	16.53 ± 7.35		

	**CARB**	21.18 ± 9.06	21.26 ± 9.55		

**Fat Mass (kg)**				.046*	.026† NOSS < PLC

	**NOSS**	14.21 ± 8.31	13.40 ± 8.10		

	**CARB**	18.64 ± 18.16	18.99 ± 18.64		

**Fat-Free Mass (kg)**				.001*	.023† NOSS > PLC

	**NOSS**	57.80 ± 8.01	59.92 ± 7.57		

	**CARB**	56.43 ± 10.33	57.02 ± 9.86		

**Upper-Body Strength (kg/kg)**				.016*	.023† NOSS > PLAC

	**NOSS**	1.03 ±0.15	1.16 ±0.21		

	**CARB**	1.07 ±0.25	1.08 ± 0.23		

**Lower-Body Strength (kg/kg)**				.001*	.035† NOSS > PLAC

	**NOSS**	4.04 ± 0.55	4.90 ± 0.64		

	**CARB**	4.19 ± 0.58	4.64 ± 0.84		

Data are presented as means and standard deviations. * Denotes a significant increase at Day 29 compared to Day 0. † Denotes a significant difference between CARB and NOSS.

### Muscle strength

For muscle strength, both groups underwent significant increases with training; however, NOSS underwent greater increases in upper-body (p = 0.023) and lower-body (p = 0.035) strength compared to CARB (Table 2).

### Serum IGF-1

Serum IGF-1 was significantly increased with training (p = 0.038); however, NOSS and CARB did not differ (p = 0.385) (Table 3).

**Table 3:** Serum and muscle markers indicative of muscle protein synthesis for the CARB and NOSS groups

Variable	Group	Day 0	Day 29	Test (p < .05)	Group x Test (p < .05)
**IGF-1 (pg/ml)**				.038*	.385

	**NOSS**	3491.53 ± 597.34	3609.63 ± 497.11		

	**CARB**	3018.43 ± 690.91	3339.24 ± 70.94		

**Myofibrillar Protein (μg/mg)**				.041*	.049† NOSS > PLC

	**NOSS**	0.089 ± 0.019	0.115 ± 0.033		

	**CARB**	0.087 ± 0.128	0.092 ± 0.022		

**MHC 1 (arbitrary density units)**				.001*	.013† NOSS > PLC

	**NOSS**	1072.93 ± 206.16	1582.37 ± 247.55		

	**CARB**	1114.95 ± 448.29	1381.76 ± 423.04		

**MHC 2A (arbitrary density units)**				.001*	.046† NOSS > PLC

	**NOSS**	904.06 ± 500.22	1502.84 ± 412.07		

	**CARB**	944.11 ± 458.98	1385.97 ± 310.87		

**MHC 2X (arbitrary density units)**				.003*	.244

	**NOSS**	878.45 ± 328.28	731.70 ± 266.26		

	**CARB**	979.89 ± 226.74	676.78 ± 163.27		

**Myo-D (Abs/mg)**				.005*	.038† NOSS > PLC

	**NOSS**	1.72 ± 0.491	2.03 ± 0.399		

	**CARB**	1.65 ± 0.339	1.74 ± 0.462		

**Myogenin (Abs/mg)**				.017*	.091

	**NOSS**	1.63 ± 0.398	1.85 ± 0.422		

	**CARB**	1.57 ± 0.240	1.78 ± 0.405		

**MRF-4 (Abs/mg)**				.001*	.001† NOSS > PLC

	**NOSS**	1.87 ± 0.236	2.25 ± 0.247		

	**CARB**	1.83 ± 0.005	1.97 ± 0.003		

Data are presented as means and standard deviations. * Denotes a significant increase at Day 29 compared to Day 0. † Denotes a significant difference between CARB and NOSS.

### Myogenic regulatory factors

All of the myogenic regulatory factors were significantly increased in both groups; however, NOSS was greater than CARB for Myo-D (p = 0.038) and MRF-4 (p = 0.001) (Table 3).

### Myofibrillar protein and MHC isoforms

Myofibrillar protein significantly increased in both groups with training (p = 0.041), with NOSS being greater than CARB (p = 0.049). All of the MHC isoforms were significantly increased in both groups with training; however, NOSS was greater than CARB for MHC 1 (p = 0.013) and MHC 2A (p = 0.046) (Table 3).

### Serum and whole blood clinical chemistry markers

Serum creatinine was significantly increased with training (p = 0.016), but was not different between groups (p = 0.413). In addition, basophils were significantly less at Day 29 for NOSS (p = 0.05). Regarding all other serum and whole blood clinical chemistry markers assessed, there were no significant changes due to training or between groups (p > 0.05), and all variables remained within normal clinical ranges throughout the duration of the study (Tables 4 and 5).

**Table 4:** Serum clinical chemistry markers for the CARB and NOSS groups

Variable	Group	Day 0	Day 29	Test (p < .05)	Group x Test (p < .05)
**Glucose (mg/dL)**				.142	.315

	**NOSS**	82.00 ± 4.98	83.00 ± 11.45		

	**CARB**	81.80 ± 6.52	86.90 ± 5.56		

**BUN (mg/dL)**				.117	.117

	**NOSS**	14.80 ± 3.42	14.80 ± 3.22		

	**CARB**	15.33 ± 2.82	12.88 ± 3.85		

**Creatinine (mg/dL)**				.016*	.413

	**NOSS**	0.896 ± 0.150	0.949 ± 0.145		

	**CARB**	0.916 ± 0.087	1.07 ± 0.201		

**Sodium (mmol/L)**				.304	.681

	**NOSS**	137.40 ± 2.79	139.72 ± 2.26		

	**CARB**	136.67 ± 2.06	137.67 ± 9.08		

**Potassium (mmol/L)**				.107	.671

	**NOSS**	4.06 ± 0.177	4.34 ± 0.568		

	**CARB**	3.95 ± 0.274	4.12 ± 0.446		

**Chloride (mmol/L)**				.665	.561

	**NOSS**	101.70 ± 2.79	103.24 ± 2.85		

	**CARB**	101.33 ± 2.64	101.11 ± 8.47		

**CO**_**2 **_**(mmol/L)**				.787	.349

	**NOSS**	21.40 ± 1.07	22.40 ± 1.07		

	**CARB**	20.11 ± 2.20	19.55 ± 4.90		

**Calcium (mg/dL)**				.799	.247

	**NOSS**	9.27 ± 0.577	9.58 ± 0.322		

	**CARB**	9.28 ± 0.481	9.07 ± 1.24		

**Protein (mg/dL)**				.914	.336

	**NOSS**	6.79 ± 0.645	6.95 ± 0.353		

	**CARB**	6.85 ± 0.657	6.65 ± 1.18		

**Albumin (mg/dL)**				.653	.244

	**NOSS**	4.41 ± 0.338	4.50 ± 0.205		

	**CARB**	4.48 ± 0.341	4.28 ± 0.695		

**Globulin (mg/dL)**				.622	.622

	**NOSS**	2.38 ± 0.410	2.45 ± 0.310		

	**CARB**	2.36 ± 0.393	2.36 ± 0.574		

**Albumin/Globulin**				.157	.436

	**NOSS**	1.89 ± 0.237	1.87 ± 0.231		

	**CARB**	1.93 ± 0.300	1.86 ± 0.304		

**Bilirubin (mg/dL)**				.181	.465

	**NOSS**	0.550 ± 0.295	0.440 ± 0.171		

	**CARB**	0.667 ± 0.282	0.633 ± 0.400		

**ALP (U/L)**				.066	.816

	**NOSS**	52.30 ± 12.84	56.40 ± 18.36		

	**CARB**	58.55 ± 15.56	63.77 ± 21.77		

**AST (U/L)**				.982	.403

	**NOSS**	17.80 ± 4.15	17.40 ± 4.67		

	**CARB**	17.55 ± 5.07	17.88 ± 4.59		

**ALT (U/L)**				.785	.785

	**NOSS**	10.70 ± 4.16	10.70 ± 6.26		

	**CARB**	7.66 ± 1.93	8.33 ± 5.63		

Data are presented as means and standard deviations. * Denotes a significant increase at Day 29 compared to Day 0.

**Table 5:** Whole blood clinical chemistry markers for the CARB and NOSS groups

Variable	Group	Day 0	Day 29	Test (p < .05)	Group x Test (p < .05)
**WBC (10**^**6**^**/L)**				.148	.300

	**NOSS**	6.06 ± 1.32	5.91 ± 1.27		

	**CARB**	7.11 ± 1.74	6.28 ± 1.34		

**RBC (10**^**9**^**/L)**				.243	.755

	**NOSS**	4.99 ± 0.304	4.94 ± 0.257		

	**CARB**	4.94 ± 0.312	4.85 ± 0.302		

**HGB (g/dL)**				.100	.695

	**NOSS**	15.24 ± 0.815	15.01 ± 0.750		

	**CARB**	14.96 ± 0.593	14.60 ± 0.563		

**HCT (%)**				.509	.997

	**NOSS**	44.28 ± 2.12	43.92 ± 2.34		

	**CARB**	43.44 ± 1.81	43.08 ± 2.03		

**MCV (fL)**				.093	.226

	**NOSS**	88.85 ± 3.90	88.90 ± 4.06		

	**CARB**	88.05 ± 4.00	88.86 ± 4.05		

**MCH (pg)**				.103	.886

	**NOSS**	30.57 ± 1.31	30.42 ± 1.34		

	**CARB**	30.32 ± 1.85	30.14 ± 1.62		

**MCHC (g/dL)**				.015	.235

	**NOSS**	34.42 ± 0.399	34.22 ± 0.482		

	**CARB**	34.45 ± 1.01	33.92 ± 1.03		

**Platelets (10**^**6**^**/L)**				.243	.861

	**NOSS**	211.24 ± 28.57	221.82 ± 38.27		

	**CARB**	228.89 ± 62.43	236.78 ± 77.51		

**Neutrophils (cells/μl)**				.178	.111

	**NOSS**	2798.63 ± 1056.71	2868.65 ± 886.18		

	**CARB**	4038.93 ± 1448.59	3263.78 ± 767.91		

**Lymphocytes (cells/μl)**				.445	.566

	**NOSS**	2512.93 ± 516.94	2344.63 ± 355.10		

	**CARB**	2164.57 ± 620.78	2139.84 ± 629.11		

**Monocytes (cells/μl)**				.613	.888

	**NOSS**	536.70 ± 121.60	509.40 ± 124.70		

	**CARB**	599.11 ± 142.53	583.67 ± 263.01		

**Eosinophils (cells/μl)**				.602	.926

	**NOSS**	186.40 ± 113.01	177.10 ± 143.54		

	**CARB**	287.33 ± 161.21	274.03 ± 183.27		

**Basophils (cells/μl)**				.293	.050† NOSS < PLC

	**NOSS**	25.70 ± 10.34	17.40 ± 8.51		

	**CARB**	21.88 ± 11.43	24.55 ± 11.10		

Data are presented as means and standard deviations. † Denotes a significant difference between CARB and NOSS.

## Discussion

The results of the present study indicate that NO-Shotgun^® ^and NO-Synthesize^® ^supplementation provided before and after resistance exercise, respectively, and in conjunction with heavy resistance training, is more effective than carbohydrate at increasing fat-free mass, muscle strength and mass, and markers of muscle protein synthesis in untrained males, while having no effect on whole blood and serum clinical safety markers. In regard to the various ingredients contained in both supplements, based on previous research it is conceivable that the primary active ingredients are whey protein, creatine, leucine, beta-alanine, and KIC.

Our rationale to use carbohydrate as a comparator was based on the premise that there is empirical evidence to suggest that carbohydrate supplementation prior to and after resistance exercise results in the maintenance of muscle glycogen [16], in addition to the fact that the insulin response associated with carbohydrate ingestion up-regulates signal transduction pathways in muscle which can activate muscle-specific gene expression and protein synthesis [17]. Therefore, many recent studies have provided nutrient provision in close proximity (either before and/or after) to resistance exercise and, in so doing, have used carbohydrate as a comparator based on the premise that carbohydrate provided in conjunction with other nutrients such as protein [17], amino acids [18], and creatine [11] has been shown to augment the responses to resistance training.

Our results demonstrated that both NOSS and CARB significantly increased total body mass (p = 0.001) with no associated increases in total body water (p = 0.345). Additionally, fat-free mass was increased in both groups (p = 0.001) with NOSS demonstrating significantly greater improvements than CARB (p = 0.023). These findings for NOSS are similar to our previous study [15] as well as a study that observed 12 weeks of heavy resistance training and creatine supplementation to induce a greater increase in fat-free mass compared to the carbohydrate group [19]. In addition, 10 weeks of heavy resistance training and whey protein and amino acid supplementation resulted in greater increases in fat-free mass compared to carbohydrate [13].

Increases in both upper- (p = 0.023) and lower-body (p = 0.035) muscle strength were significantly greater in NOSS compared to CARB. The present data are corroborated by our previous study [15], along with previous other studies which have demonstrated heavy resistance training, when combined with creatine [9,19] whey and casein protein and amino acids [13], and whey protein and amino acids [12] produces greater increases in muscle strength.

Our present results demonstrated that NOSS supplementation results in preferential increases in myofibrillar (p = 0.049) protein and Type I (p = 0.013) and IIa MHC (p = 0.046) when compared to carbohydrate. Our results are similar to a study in which creatine supplementation in conjunction with 12 weeks of resistance training resulted in an increase in myofibrillar protein and MHC isoform content when compared to carbohydrate [19]. Additionally, a protein and amino acid supplement ingested in concert with 10 weeks of heavy resistance training induced a greater increase in myofibrillar protein compared to carbohydrate [13].

As with our previous study [15], we observed serum IGF-1 to be increased with heavy resistance training after four weeks; however, there was no difference between groups (p = 0.385). Previous studies have demonstrated heavy resistance training to either increase [20] or have no effect [21] on serum IGF-1. We have previously shown that 10 weeks of heavy resistance training combined with a daily supplement containing whey/casein protein and free amino acids increased circulating IGF-1 levels [13]. Even though serum IGF-1 was increased in the present study, we can conceivably conclude that none of the ingredients contained in the supplements ingested by both groups served as IGF-1 secretagogues. Even so, this outcome may not be germane to the results as hepatically-derived circulating IGF-1 appears to have no direct effect on muscle hypertrophy [22] compared to skeletal-muscle derived IGF-I which has been shown to increase in response to resistance training [23] and induces muscular protein synthesis [24,25].

The MRFs (Myo-D, myogenin, MRF-4, myf5) are transcription factors that play a role in muscle hypertrophy by binding to E-boxes in the promoter region of various sarcomeric genes [4], thereby up-regulating transcription which can invariably lead to an increase in protein synthesis. It appears that myogenin and MRF-4 specifically up-regulate the expression of genes specific to contractile protein [26,27] and fast and slow muscle fiber differentiation [28]. Type I and II muscle fibers have been shown to preferentially accumulate myogenin and Myo-D, respectively [26]. Creatine supplementation in conjunction with resistance training has been shown to increase MyoD, myogenin, and MRF-4 that were correlated with increased MHC and myofibrillar protein [29] and myofiber size [30]. In line with our previous studies, the present results demonstrated that both groups underwent significant increases in MRF content [15,29] and all three MHCs [15,19]. However, NOSS underwent even greater increases in Myo-D (p = 0.038) and MRF-4 (p = 0.001) and Type 1 and 2A MHC. It is difficult to conclude which specific ingredient elicited these results; however, based on previous research we can speculate a role for creatine since 12 weeks of supplementation and heavy resistance training resulted in muscle hypertrophy, along with concomitant increases in MHC Type 1, 2A, and 2X protein, and myofibrillar protein [19].

As with most nutritional supplements, NO-Shotgun^® ^and NO-Synthesize^® ^are comprised of a number of different compounds with most having no little, if any, clinical safety data available. During the course of the study, we observed no significant changes beyond the normal clinical ranges in regard to clinical safety measures in either group. These data indicate that the ingestion of carbohydrate, NO-Shotgun^®^, and NO-Synthesize^® ^for a period of 28 days has no detrimental clinical effects with regard to the whole blood and serum variables assessed.

A purpose of the present study was to compare the effects of NO-Shotgun^® ^given pre-exercise and NO-Synthesize^® ^given post-exercise to our previous study (15) in which only NO-Shotgun^® ^was given pre-exercise to determine if additional post-exercise nutrient provision would provide an augmented effect. Our present data showed that total body mass to increase by 1.51% and fat-free mass to increase 3.66% in the NOSS group. This mirrors the results observed in our previous study in which the NO-Shotgun^® ^(NO) group increased 2.59% and fat-free mass 4.75% (15). In our previous study, upper- and lower-body strength increased 8.82% and 18.40%, respectively, with only lower-body being greater than placebo (15). However, our present results show a preferential increase of 12.62% and 21.28%, respectively, for upper- and lower-body strength with both being greater in NOSS. In our previous (15) and present study, serum IGF-1 increased 9.34% and 3.38%, respectively, with neither being different from placebo. Myofibrillar protein was preferentially increased by 70.39% in the NO group in our previous study (15) and 29.21% in our present study. For the MRFs, in our previous study, they were preferentially increased in the NO group by 70.91%, 56.24%, and 71.17% for Myo-D, MRF-4, and myogenin, respectively. While still preferentially increased in the NOSS group in our present study, Myo-D, MRF-4, and myogenin increased 18.02%, 20.32%, and 13.49%.

NO-Shotgun^® ^and NO-Synthesize^® ^contain a proprietary blend of a number of compounds assumed to be effective at increasing muscle strength and mass such as creatine, arginine, glutamine, beta-alanine, keto-isocaproate (KIC), and leucine, casein and whey protein, branched-chain amino acids, lysine, phenylalanine, threonine, histidine, and methionine. As a result, attempting to isolate which specific ingredient has the greatest impact on our outcome measures is not feasible. However, our present results indicate that supplementation protocol of providing NO-Shotgun^® ^pre-exercise, NO-Synthesize^® ^post-exercise, and NO-Synthesize^® ^on non-exercise days for 28 days is more effective than carbohydrate at increasing muscle mass and strength and markers indicative of muscle protein synthesis, while having no negative impact on the clinical chemistry variables assessed. Furthermore, our present results demonstrate preferential improvements in muscle strength and mass and agree with our previous study [15], and suggest that nutrient provision before and after resistance exercise is effective in preferentially augmenting muscle strength and mass.

## Competing interests

This study was supported by an independent research grant from VPX (Davie, FL) awarded to Baylor University. DSW has previously served as a paid consultant for VPX; however, he was not serving in this capacity during the time in which this study was being conducted, and has no financial interests concerning the outcome of the investigation. The authors declare that they have no competing interests.

## Authors' contributions

All researchers involved independently collected, analyzed, and interpreted the results from this study. MS assisted in coordination of the study, data acquisition, in performing the statistical analysis, and drafting the manuscript. NS, SL, TC, MM, and VL participated in the data acquisition. DSW conceived the study, developed the study design, secured the funding for the project, assisted and provided oversight for all data acquisition and statistical analysis, assisted and provided oversight in drafting the manuscript, and served as the faculty mentor for the project. All authors have read and approved the final manuscript.
